# Report of two cases of Accessory Cavitated Uterine Mass (ACUM): Diagnostic challenge for MRI

**DOI:** 10.1016/j.radcr.2021.07.071

**Published:** 2021-09-07

**Authors:** Mélanie Mollion, Aline Host, Emilie Faller, Olivier Garbin, Raluca Ionescu, Catherine Roy

**Affiliations:** a Department of Radiology B, Strasbourg University Hospital - New Civil Hospital, Strasbourg, 67091 Cedex, France; b Department of Gynecology, Strasbourg University Hospital - Hautepierre Hospital, Strasbourg, 67200 Cedex, France; c Department of Gynecology, Strasbourg University Hospital - Obstetric Medico-Surgical Center (CMCO), 67303 Schiltigheim Cedex, France

**Keywords:** MRI, Adenomyosis, Müllerian anomaly, Transvaginal Ultrasound, Juvenile cystic adenomyoma. Accessory Cavitated Uterine Mass. Laparoscopy

## Abstract

Cystic adenomyosis is an unusual form of adenomyosis, characterized by a well-circumscribed cavitated endometrial gland and stroma, ≥ 1 cm in diameter, located within the myometrium. Few cases have been reported in the gynecological literature, with confusing naming such as: juvenile cystic adenomyosis, cystic myometrial lesions, cystic adenomyoma or juvenile adenomyotic cysts. The current preferred terminology is accessory cavitated uterine mass /or malformation (ACUM). We report here the cases of two 17 and 18 -year-old nulliparous women, who complained of severe dysmenorrhea early after the onset of menarche, with none or partial efficiency of medical treatment. MRI findings, with a follow-up in one case and surgical treatment in both cases, are described with an emphasis on physiopathology. The typical MR appearance is a large well-circumscribed round mass within the external myometrium, composed by an inner cystic hemorrhagic layer surrounded by a thick fibrous crown. The first-line treatment is laparoscopic surgery with mass resection. This typical MRI pattern must be a part of the knowledge of the radiologists.

**Abbreviations**
MRI: Magnetic resonance imaging
ACUM: Accessory Cavitated Uterine Mass

## Introduction

As stated by Bird [1] in 1972, ‘Adenomyosis may be defined as the benign invasion of endometrium into the myometrium, producing a diffusely enlarged uterus which microscopically exhibits ectopic non-neoplastic, endometrial glands and stroma surrounded by the hypertrophic and hyperplastic myometrium’.

It may be focal or, more frequently, diffuse with several cystic spaces filled with blood, usually small, 5 mm in greatest diameter and typically located within the junctional zone.

The cystic form is an unusual finding characterized by a unique larger hemorrhagic cyst lined by endometrium and located within the myometrium. The physiopathology of this entity is incompletely understood, as its MR appearance. We report two cases with a typical MR imaging pattern and surgical and pathological correlation.

## Cases

Case 1: (Fig. 1)

**Fig. 1:**
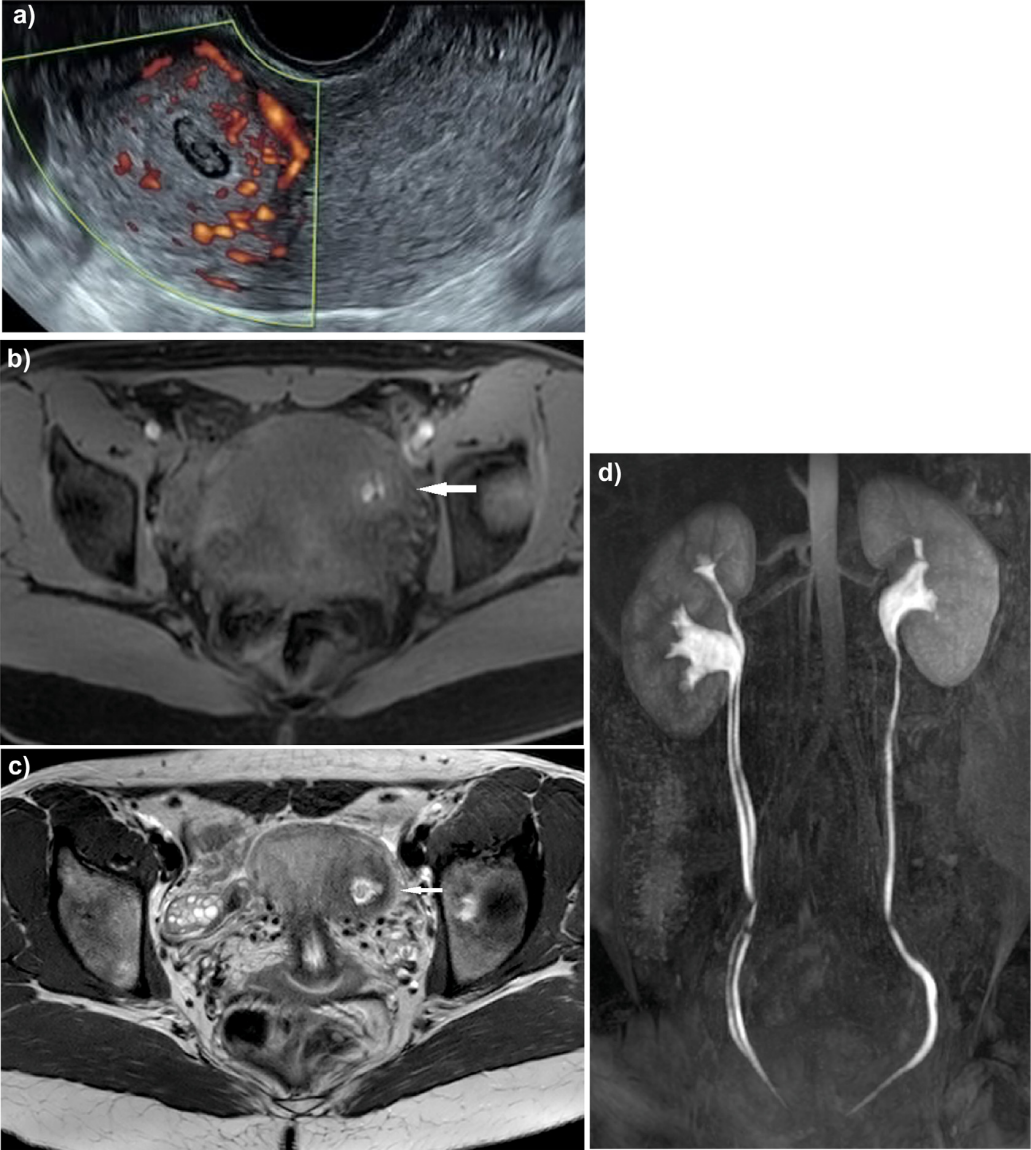
17-year-old nulliparous girl with intense dysmenorrhea since the age of 16 years. a: Transvaginal ultrasound with Doppler revealed in heterogenous hypoechoic meaning the presence of blood clots in the left part of the myometrium. It was surrounded by vascularization. b: T2-weighted sequence - axial view. The lesion was located in the left part of the uterus near the origin of the round ligament (↑white arrow). The heterogenous inner part indicated the presence of blood clots. The ovaries were normal (only the right one is visible in this slice). c: T1-weighted sequence – axial view. The hypersignal inside the inner part of the llesion indicated the presence of blood (↑white arrow). d: MR Urography-coronal view. A minor congenital urinary tract abnormality was present: duplication of the right urinary system. (Color version of the figure is available online.)

This 17-year-old nulliparous girl presented disabling dysmenorrhea and abdominal cramping, leading to several hospitalization. Her first menses occurred at the age of 12 years, and her menstrual cycle was more or less regular. She was occasionally sexually active and no contraceptive pill was taken.

In the past year, the pain had become more severe and refractory to any analgesic or non-steroidal anti-inflammatory drugs. There was no dyspareunia or dyschezia. Biological routine tests including complete blood cell count, urinary analysis and erythrocyte sedimentation rate were within normal limits. CA 15.3, carcinoembryonic antigen, and b-human chorionic gonadotropin levels were normal, whereas CA 125 level was slightly elevated (40U/mL).

The pelvic examination found a painful mass in the left part of the pelvic cavity.

Transvaginal ultrasound revealed a well-circumscribed mass along the external part of the myometrium with a heterogeneous hypoechoic center surrounded by an annular vascular thick rim (Fig 1a).

On Pelvic MRI (Figs. 1b and c), performed on a 3T unit, the mass appeared as a well delineated rounded lesion of 30mm. It was intra-myometrial and located in in the left uterine horn, at distance from the endometrial cavity, with heterogeneous hypersignal in the T1-weighted sequence corresponding to a hemorrhagic content with clots, surrounded by a thick fibrous hypointense crown in the T2-weighted sequences. There was no other sign of endometriosis elsewhere in the pelvic cavity. A minor congenital abnormality of the urinary tract was also found.

Laparoscopic surgery was undertaken. The mass was found in the uterus close to the left round ligament. Aspiration of chocolate-colored fluid from the cyst was done before its radical excision after an easily finding of correct surgical planes. The pathological examination confirmed the diagnosis of hemorrhagic cystic lined with endometrial glands and stroma surrounded by smooth muscle hyperplasia, consistent with a cystic adenomyoma.

Case 2: (Fig. 2)

**Fig. 2:**
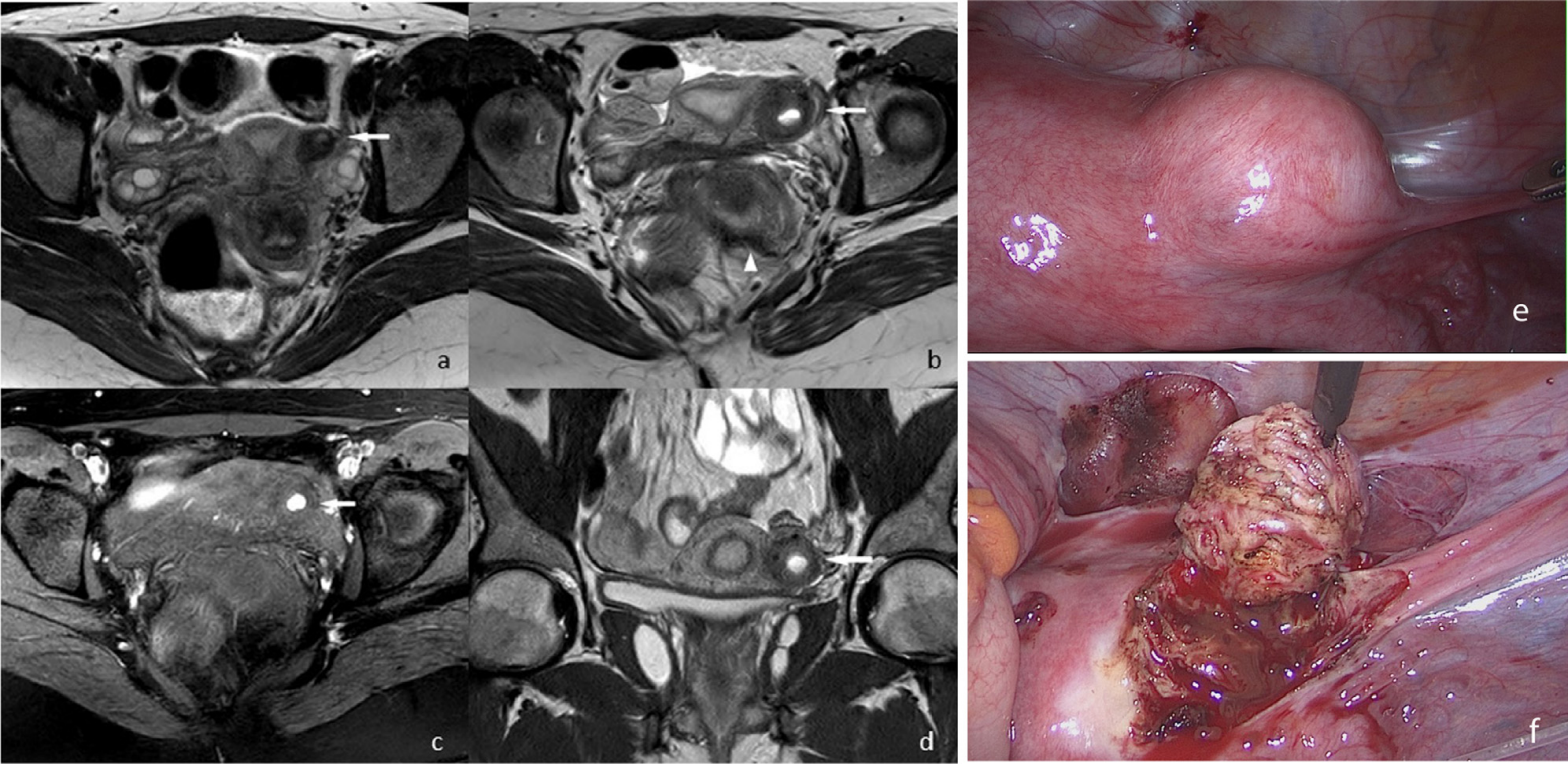
A 23-year-old nulliparous young woman referred for chronic major dysmenorrhea. a: T2-weighted seque ce - axial view. First examination performed in 2015. The lesion is small (15 mm) with a small inner cystic part surrounded by a hypointense annular tissue. b: T2-weighted sequence - axial view. Second examination performed in November 2020. The lesion had increased in size, now measuring 30mm. The hyperintense inner part presented a fluid-fluid lesion meaning different age for the blood. The lesion is located in the left part of the uterus near the origin of the round ligament (↑white arrow). The heterogenous inner part indicated the presence of blood clots. It was also found a thickening of the inner part of the left uterosacral ligament (▲white arrow head) c: T1-weighted sequence – axial view. The hypersignal inside the inner part of the lesion is larger than previously (↑white arrow). d: T2-weighted sequence -coronal view. It demonstrates the precise localization of the lesion at the level of the left uterine horn (↑white arrow). e: View of the laparoscopic surgery performed in February 2021. Intraoperative appearance of the mass located on the left side of the uterus and extending toward the left side of the broad ligament with smooth border.f: View of the laparoscopic surgery performed in February 2021. After incision, the chocolate-colored fluid was drained and the lesion was excised from surrounding myometrium. (Color version of the figure is available online.)

A 23-year-old nulliparous young woman was referred to our department due to long standing chronic major dysmenorrhea and dyspareunia, with no response to analgesic drugs. She had no previous medical story. Her first menses occurred at the age of 13 years. She localized the complaint mainly in the left part of the pelvic cavity, starting one week before the onset of menses and disappeared 2-3 days later.

A first MRI performed in 2015 revealed a 15 mm rounded formation of the left uterine horn, with an intra-myometrial topography, distinct from the endometrial cavity, with a central hypersignal T2-weighted surrounded by a thick hypointense fibrous crown (Fig. 2a). No pertinent diagnosis was done at that time and the patient received a prescription for a cyclical oral contraceptive drug, and was lost for follow-up at that time.

She was re-referred in November 2020 for disabling dysmenorrhea and induration of the left uterosacral ligament on clinical examination. The intensity of the pain was becoming progressively very intense without efficiency of the medical treatment. A second pelvic MRI examination found an increased in size lesion measuring 30 mm, with a larger central fluid and a hypointense crown (Fig. 2b). It was also found a thickening of the internal part of the left uterosacral ligament in favor of an associated of a posterior subperitoneal endometriosis. There was no abnormality of the ovaries.

The surgical exploration revealed a round 3 × 3-cm uterine mass arising from the left lateral uterine wall and extending toward the left side of the broad ligament with smooth border. Small foci of endometriotic lesions were also noted on the left uterosacral ligament. The external layer of the mass was incised, and chocolate-colored fluid was drained. The appearance of the tissue lining the cyst wall was similar to that of a normal endometrium. The cyst wall was then enucleated, leading to its complete resection from the surrounding myometrium (Figs. 2e and f). The pathological examination revealed a central cavity lined with functional endometrium and surrounded by smooth muscle and confirmed the diagnosis.

## Discussion

Adenomyosis of the uterus is a very common imaging finding [2] whereas the large cystic isolated form presented in our two cases is a rare and most likely underdiagnosed condition.

Cystic lesions within the myometrium are uncommon. They are classified into two main groups: congenital and acquired. Acquired cysts include the cystic degeneration of uterine leiomyoma, the cystic adenomyosis and serosal cysts. Congenital cysts derive from Mullerian duct anomalies such as a noncommunicating rudimentary horn and a unicornuate uterus.

The clinical context is very suggestive for adenomyosis. This particular cystic form occurs in adolescents and women younger than 30 years, with intense dysmenorrhea appearing shortly after puberty and more or less resistant to different medical treatment. This severe manifestation can be attributed to the recurrent intra-cystic bleeding producing a distension of the cystic cavity.

Few cases have been published mainly by gynecologists and using various terms, such as juvenile cystic adenomyosis, adenomyotic cyst, cystic adenomyoma, and cystic adenomyosis [3]. There is no agreement on the pathogenesis of this process.

Several explanations have been raised [4,5].

An attractive hypothesis was proposed by Takeda et al [6] who suggested that this hemorrhagic cyst is due to a congenital anomaly such as a non-communicating accessory cavity type lined with endometrial epithelium due to the persistence of Mullerian duct tissue. It has been named juvenile cystic adenomyosis. There are several arguments in favor of this origin: the very early onset of severe post pubertal symptoms, the age of onset under 20 years, the intra-myometrial topography close to the origin of the round ligament and far from the endometrium, the absence of communication with the endometrial cavity, the size up or equal to 1 cm, its isolated character without any other sign of endometriosis and the frequency of associated birth defects of the urogenital tract [7,8], as in our case 1.

By considering patient age at symptom occurrence (younger or older than 30 years), some authors have classified these lesions into 2 categories: the adult and the juvenile form. The adult form seems to be the result from a traumatism at the endometrium-myometrium interface, e.g., after uterine instrumentation [9].

The last hypothesis consider that these two forms have the same underlying process and are related to a cystic variant of adenomyosis, sometimes associated with another localization of endometriosis or adenomyosis [10].

In the recent literature, the term of ACUM (Accessory Cavitated Uterine Malformation or Mass) is now preferred to the others previously cited. There are several arguments for this terminology: variable age of patient, constant appearance and localization in contrast to the multiple forms of adenomyosis. For Naftalin [11], the term ‘malformation’ should be used instead of mass as it reflects a benign lesion.

However, at histological analysis, all those formations present the same pattern. This is a cavity lined by an endometrial epithelium. The stroma below the epithelium is thin throughout the cyst and contains red blood cells and hemosiderin-laden macrophages. The stromal cells are morphologically similar to those of the endometrium, as in endometriosis. It is surrounded by a thick myofibroblastic wall corresponding to the reaction of the myometrium to menstrual bleeding as seen on imaging findings.

Although the first-line imaging is endovaginal ultrasound, taken full advantage using 3 -D modality which shows an intra-myometrial rounded cystic image, with sharp edges and finely echogenic fluid content, its appearance is more characteristic on MRI. The advantage of MRI is to provide a precise localization and morphological aspect, a tissue characterization of the lesion as well as an evaluation of the overall pelvic cavity. There is no need for any contrast injection. An hypersignal on T1 and T2-weighted sequences reflects a hemorrhagic content and hypointensity of the surrounded tissue on T2-weighted is the typical aspect of the fibrous tissue. A liquid-liquid level can be observed within the cyst, corresponding to a bleeding content of different ages.

In agreement with the recent paper of Peyron [12], the lesion of our cases was left-sided and within the external myometrium under the horn of the round ligament insertion. We found a minor urinary malformation in case 1 and endometriosis on uterosacral ligament in case 2. In addition, the follow up in case 2 indicates that the lesion can slowly grow over time. MRI findings may also be useful in selecting the right approach for the surgical treatment.

In addition, MRI provides some arguments for the differential diagnosis. A congenital anomaly with hematometry within a non-communicating uterine horn will have an oval or tubular configuration without any surrounded fibrous layer. The necrobiosis of a uterine myoma will have a different topography, also containing a T2-weighted hypersignal in but a thinner hypointense wall on T2-weighted sequences without enhancement, at the difference of ACUM if an injection is performed [13, 14].

Several types of invasive management are possible, without consensus; since medical treatment does not work in the long term. Surgical adenomyomectomy has the disadvantage of surgical risk and weakening of the uterus. Interventional radiology techniques using cryotherapy or focused ultrasound are under evaluation.

In conclusion,This uncommon uterine abnormality called ACUM has a so typical appearance on MRI, allowing the right diagnosis as well as a complete evaluation of the pelvic cavity. This feature must be a part of the knowledge of the radiologists.

## Patient consent

Case report *of Two Cases of Accessory Cavitated Uterine Mass (ACUM): diagnostic challenge for MRI* I confirm that our 2 patients have been informed that their case has been proposed for publication in a scientific imaging journal. I confirm that written, informed consent for publication of their case was obtained from the patients. Our local Institutional Review Board, was orally informed of this work and had approved these data.
